# Current Insight of Collagen Biomatrix for Gingival Recession: An Evidence-Based Systematic Review

**DOI:** 10.3390/polym12092081

**Published:** 2020-09-13

**Authors:** Ruth Naomi, Retno Ardhani, Osa Amila Hafiyyah, Mh Busra Fauzi

**Affiliations:** 1 Centre for Tissue Engineering and Regenerative Medicine, Faculty of Medicine, Universiti Kebangsaan Malaysia, Cheras, Kuala Lumpur 56000, Malaysia; ruthmanuel2104@gmail.com; 2 Department of Dental Biomedical Sciences, Faculty of Dentistry, Universitas Gadjah Mada, Jl Denta Sekip Utara, Yogyakarta 55281, Indonesia; retnoardhani@mail.ugm.ac.id; 3 Department of Periodontics, Faculty of Dentistry, Universitas Gadjah Mada, Jl Denta Sekip Utara, Yogyakarta 55281, Indonesia; osaamilahafiyyah@ugm.ac.id

**Keywords:** collagen, gingival recession, biomaterial, healing mechanism, tissue regeneration

## Abstract

Collagen (Col) is a naturally available material and is widely used in the tissue engineering and medical field owing to its high biocompatibility and malleability. Promising results on the use of Col were observed in the periodontal application and many attempts have been carried out to inculcate Col for gingival recession (GR). Col is found to be an excellent provisional bioscaffold for the current treatment in GR. Therefore, the aim of this paper is to scrutinize an overview of the reported Col effect focusing on in vitro, in vivo, and clinical trials in GR application. A comprehensive literature search was performed using EBSCOhost, Science Direct, Springer Link, and Medline & Ovid databases to identify the potential articles on particular topics. The search query was accomplished based on the Boolean operators involving keywords such as (1) collagen OR scaffold OR hybrid scaffold OR biomaterial AND (2) gingiva recession OR tissue regeneration OR dental tissue OR healing mechanism OR gingiva. Only articles published from 2015 onwards were selected for further analysis. This review includes the physicochemical properties of Col scaffold and the outcome for GR. The comprehensive literature search retrieved a total of 3077 articles using the appropriate keywords. However, on the basis of the inclusion and exclusion criteria, only 15 articles were chosen for further review. The results from these articles indicated that Col promoted gingival tissue regeneration for GR healing. Therefore, this systematic review recapitulated that Col enhances regeneration of gingival tissue either through a slow or rapid process with no sign of cytotoxicity or adverse effect.

## 1. Introduction

### 1.1. Gingival Recession (GR) 

**Figure 1:**
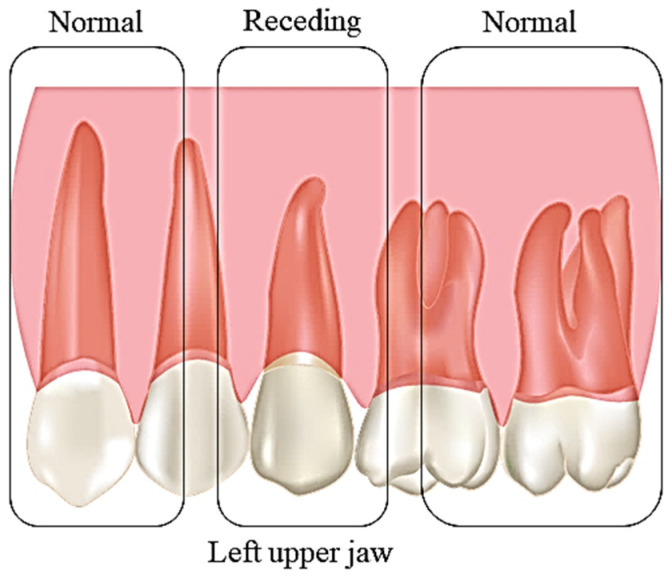
Comparison of normal gum and gingival recession.

GR is commonly known as receding gums, in the apical margin of the gingiva migrates to the cementoenamel junction. The extent of the recession is defined based on the space between the margin of the gingiva and the cementoenamel junction [1,2]. It usually affects middle-aged group population. The condition can be categorised as localised or generalised depending on the severity of the condition [3]. The severity of the condition can be further classified according to the Miller classification system comprising of class I, II, III, and IV [2,4] or Atkin and Sullivan classification on the basis of the width and depth (shallow-narrow, shallow-wide, deep-narrow, or deep-wide) [5]. There are a few factors that closely resemble the occurrence of GR including age, malposition and imperfect tooth brushing techniques, abnormal frenal attachment, occlusion resulting in trauma, inflammation of the gingiva, masochistic habits, smoking, and the presence of calculus. GR can be termed as non-acute and progressive over time. The symptoms are usually unnoticeable as only minimal changes occur over the days (Figure 1). The notable symptoms are exposed roots, loose and longer teeth, gum bleeding, the formation of plaque, caries, and hypersensitivity [6,7,8]. According to the Ministry of Health Malaysia, gum disease has been categorised as one of the major global dental problems. In Malaysia, the prevalence of gum disease is very high, affecting nearly 14.4 million or 94% of the Malaysian population [9]. In contrast, in Indonesia, this problem affected only 14% of the total population [10]. According to the statistic in Malaysia, only 1 out of 10 people has a healthy gum, while the remaining tend to have gum-related disease [9]. In order to accommodate this problem from worsening, recent advancement in biomaterials is considerable as it has high biocompatible properties to human nature. Many studies have used Col-based scaffolds to study its effects in gingival healing using in vitro models [11,12], in vivo models [13,14,15,16], and clinical trials [17,18,19,20,21,22,23,24,25].

### 1.2. Collagen (Col)

Collagen is the most abundant of the types of protein that make up the extracellular matrix in mammals [26,27]. Col is a fibrous structured protein comprising 25% of the total protein content in humans [28], in which 99% of Col type I and III and 1% of Col type IV make up the gingival connective tissue (CT) [29]. To date, there are 25 different types of Col that have been identified, comprising of 46 variations in the polypeptide chains [30]. Col is made up of a triple helix, which is twisted into a rope-like structure [26,31]. The triple helix structure is stretch-resistant to ensure that the Col is inextensible [28]. The triple helix structure mainly comprises two α1 chains that are identical with the α2 at the third position, which influences the chemical composition of the Col. It comprises repeating units of three amino acids of Glycine-X-Y [32,33,34] Glycine molecule ensures the packaging of 3α, units together forming a tropocollagen. To form a fibrillar, Col is then packed into certain defined shapes, including hexagonal and quasi hexagonal, that appear microscopically as elongated fibrils microstructure [30,35]. Col is also the main component in the skin, bones, teeth, cartilage, tendons, and ligaments owing to the tensile strength. Being the main component in the periodontal ligament (80%), the functions of Col are to maintain the structure and the mechanical strength [28,36], besides acting as a protective barrier to prevent the absorption of pathogens and toxins [14]. Col is synthesised twofold lower in the gingival tissue compared with the skin [37]. Furthermore, Col type III makes up as the major component for granulation tissue in the gingival structure [38]. However, upon maturation, Col reduced to 20% of the total Col content [29]. During the healing phase, particularly for gingival, Col deposition will exceedingly increase to support the rapid healing as a natural mechanism [38,39]. However, in the final healing phase, Col formation will decrease gradually. This is to ensure a controlled rate of angiogenesis to promote the migration of endothelial cells towards the injured gingival tissue [29].

### 1.3. Collagen for Periodontal Tissue Regeneration 

Collagen is a natural component in human and has shown promising results as a biomaterial for tissue regeneration [40]. Periodontal tissue comprises heterogeneous cell populations in a complex structure to support the tooth growth [41]. The primary stage of tissue regeneration in periodontal is initiated by the migration and proliferation of the periodontal ligament [39]. The currently available treatment aims to stimulate the release of growth factors to enhance the migration and proliferation of the periodontal ligament, thereby enhancing the healing cycle [41]. Kim et al. demonstrated that Col sponge has the potential to absorb the growth factors, hence contributing to the rapid regeneration of the periodontal tissue [42]. Apart from this mechanism, Col is also used to prevent the migration of the epithelial cells owing to its capacity to act as a barrier [43]. Therefore, Col permits the cells with regenerative ability to occupy the defected region in the oral tissue [44]. In this process, Col is able to restore the normal function of the damaged tissue by promoting regeneration, thus salvaging the aesthetic issue [8]. As Col naturally possesses high malleability, weak immunogenicity, and haemostatic, it can be easily integrated with any specific tissue type [44]. Upon introducing Col scaffold to the oral tissue, the fibril will bind to the native tissue causing tissue augmentation [8]. Furthermore, gingival tissue has been identified as a source of mesenchymal stem cell (MSC) derivative and is easily isolated with high proliferation rate characteristics [45] upon being introduced to Col scaffold [46]. Meanwhile, dental-derived MSC has been classified as the best alternative for regenerative medicine, specifically in the dentistry. It naturally possesses the immunomodulatory characteristics of phytohemagglutinin (PHA)-stimulated peripheral blood mononuclear cells (PBMCs) proliferation. Thus, fibroblast isolated from the gingival tissue has been proven to possess the same property as the dental-derived MSC. This characteristic is more prone to be noted in the local environment of the oral region [47,48].

### 1.4. Cell–Biomaterial Interaction 

**Figure 2:**
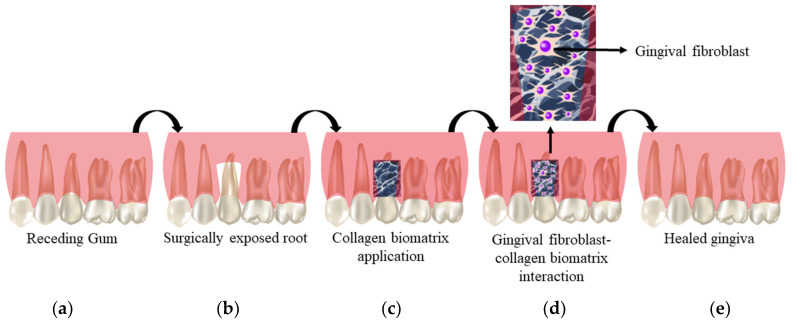
Cell biomaterial interaction: (**a**) receding gum; (**b**) exposed region of the gum; (**c**) collagen scaffold application; (**d**) mechanism of action of collagen biomatrix; (**e**) healed gingiva.

The interaction between the cells and the biomaterial is defined through the degree of cells adhesion towards the biomaterial. Particularly for gingival regeneration, cell adhesion in the Col scaffold greatly influences the migration, integration, and survival of the cells within the scaffold (Figure 2). The interaction will be achieved by incorporating one or more fragments of the native tissue into the scaffold [49]. In this scenario, the expression of Col type I (Col-I) in healthy gingival tissue is common [50]. The characteristic of Col as the natural component in human gingival tissue also eases the application of Col biomatrix for GR. Col, being a chemoattractive agent for fibroblasts, will eventually enhance the attachment of gingival fibroblast towards Col scaffold. It can be hypothesized that the cell attachment will further stimulate the spreading of the cells in the scaffold causing dilation of the central lumen of the fibroblast [51]. This then will expose the root surface in the GR to attach with the new connective tissue, thus eliminating the need for further surgery to treat GR. Being highly biocompatible molecules, Col scaffold also serves as a haemostatic agent at the affected region in the periodontal. The Col biomatrix will stimulate the clot formation at the early stage to stabilise the wound. This is preceded by attracting the fibroblast to enhance the primary wound closure. When this occurs, Col biomatrix augments the thickness of the gingival tissue. Nevertheless, Col biomatrix is permeable to oxygen, and thus will further ease the flap healing by permitting an adequate exchange of gas and nutrients at the affected gingival region [52].

### 1.5. The Role of Pre-Clinical Investigation and Safe Approach of Collagen Biomatrix for GR

In this era, the utilisation of biomaterial and biomatrices has gained much attention [53]. Specifically, for GR, the potential of biological regeneration is very high for Col biomatrix. Pre-clinical studies have proven that Col is highly biocompatible for periodontal application, and thus serves as a baseline for human clinical trials. Researchers have proven that biomaterials are effective in obtaining clinical attachment and probing pocket-depth reduction. Pre-clinical studies have also further contributed to the evolution of new regenerative techniques for GR healing. One of the evolutions includes the transformation from large flaps procedure to minimally invasive techniques, which in turn reduces the risk of high mortality rate owing to the modern surgical procedure [54]. Meanwhile, the safe approach and controlled quality also enhance the safe application of biomaterial or biomatrices for humans. In this case, highly efficient reparative techniques are much encouraged as the oral cavity has constant contact with the environment, especially while eating or communicating. Similarly, an adequate amount of supplemented medium influences the outcome as an ideal biomaterial for periodontal application. This is to ensure good cell viability and proliferation within the scaffold upon application [55]. To ensure the safe application of biomaterials in human models, the Food and Drug Administration (FDA) guidelines must be strictly followed, particularly for the source and procedure of Col derivative. Furthermore, the Col biomatrix utilisation for clinical trial must be handled with care and the safety protocol application and the serum-free medium is also highly encouraged [56].

## 2. Materials and Methods 

### 2.1. Search Strategy 

This review was conducted following the preferred notification items for systematic reviews and meta-analyses (PRISMA) [57]. A systematic review for the literature search was performed to identify relevant articles on the usage of a Col-based scaffold for GR. A literature search was performed comprehensively in a few selected databases such as EBSCOhost, ScienceDirect, Springer Link, and Medline & Ovid. The literature search was strictly selected to any publications related to the topic from 2015 onwards. The search query was chosen based on the Boolean operators, as described elsewhere [58]. It involves the use of two sets of keywords, including (1) collagen OR scaffold OR hybrid scaffold OR biomaterial AND (2) gingiva recession OR tissue regeneration OR dental tissue OR healing mechanism OR gingiva.

### 2.2. Inclusion Criteria 

Only original articles were selected to be reviewed including studies on in vitro, in vivo, and clinical trials that primarily discussed the characteristics, biocompatibility, and outcome measures of collagen bioscaffold for GR. Articles discussing GR based on Miller’s classification (Miller’s class I, II, and/or III) were only selected for further analysis. The chosen articles must be written in the English language with abstracts. The selected studies must evaluate at least the following essential measures such as (1) the physicochemical characteristics of the Col-based scaffold, (2) the response exhibited by the subject upon implanting the scaffold, (3) the positive or negative outcome upon implanting the scaffold, and (4) GR based on Miller’s classification (Miller’s class I, II, and/or III). The reference search is limited within the last 6 years of publication, starting from 2015 until July 2020.

### 2.3. Exclusion Criteria 

All secondary literature, case reports, patents, thesis dissertations, original papers without abstracts, and articles written in any other language than English have been automatically excluded from being analysed. Besides, papers that did not fulfil the inclusion criteria were also excluded. The studies focusing on Col scaffold for any other periodontal disease other than GR were also excluded from this review.

### 2.4. Data Extraction and Management

The screening process of all articles in this review paper was performed according to the listed inclusion criteria. The titles and abstracts of each article were screened ensure the article meets the inclusion criteria. There were three independent reviewers (R.N., R.A., and M.B.F.) responsible for screening the titles and the abstracts of all identified records for potentially relevant studies. The records that were chosen were further reviewed by reading the full-text to ensure eligibility. Any disagreement was discussed between the authors and, whenever necessary, a fourth reviewer (O.A.) was consulted. Further screening covered the full text of articles that met the inclusion criteria before the next step of the data extraction process. Data extraction was performed using the standardised data extraction form based on the guideline to ensure the quality of the study and for evidence synthesis. The recorded information consists of (1) the aim of the study, (2) the study design, (3) the subject type, (4) findings of the parameter assessed, and (5) the final outcomes of the study. The characteristics and findings assessed are more generalised rather than a primary outcome in the respective study. Meantime, no duplication nor dispersion measures were described for in vitro and in vivo study. Regarding that, no bias was reported for in vitro and in vivo study, while for clinical trials, bias assessment was done.

### 2.5. Strategy for Data Extraction

A systematic review of the findings from the selected studies is provided. It is structured to ensure the characteristics of the scaffold and the study outcomes are described in the Results section. The main extracted information is extensively described in the Results section. In the Discussion section, the analysis of the results is presented considering the suitability of the Col scaffold for GR with some suggestions for future applications in standardised approaches.

### 2.6. Risk of Bias Assessment 

**Figure 3:**
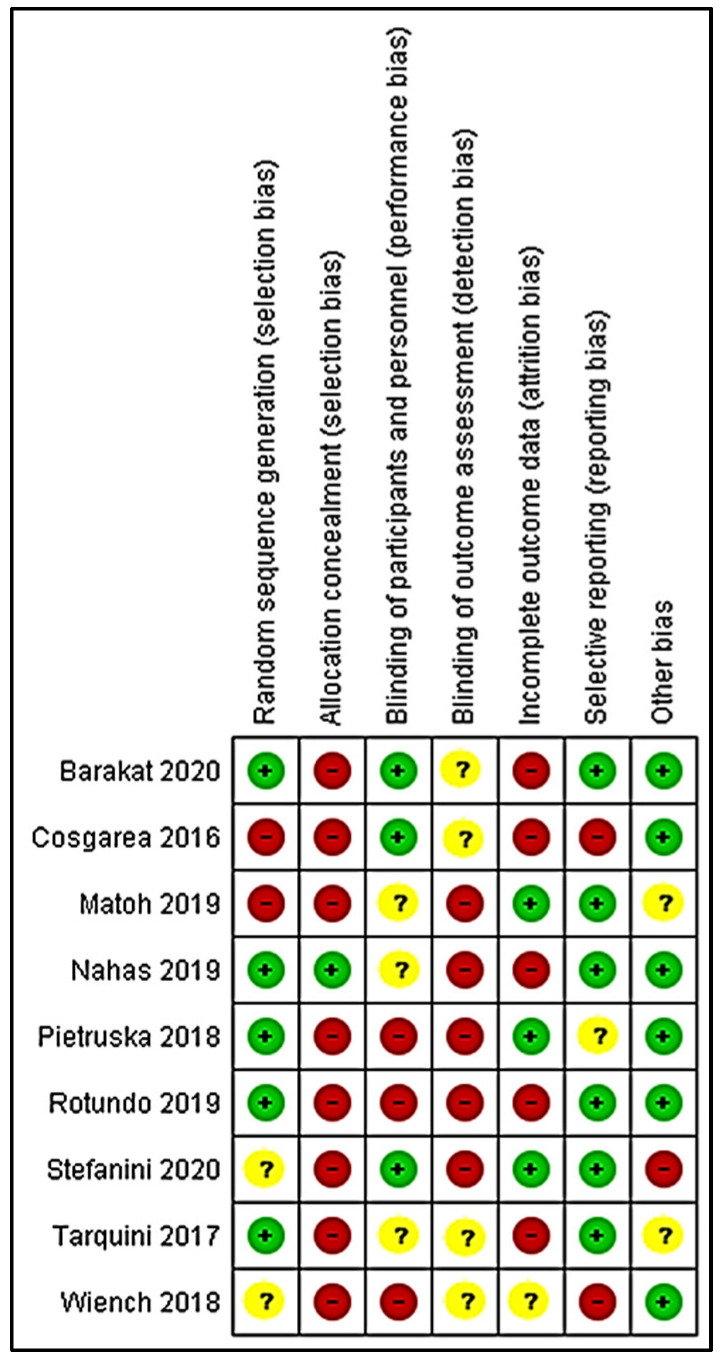
Risk of bias assessment for clinical trial.

**Figure 4:**
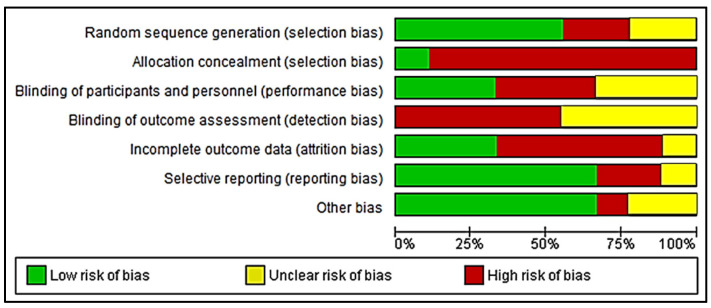
Risk of bias graph.

Three reviewers (R.N., R.A., and M.B.F.) evaluated the risk of bias of the included studies using an adapted revised version of the Cochrane risk-of-bias tool for randomized trials (RoB 2) [59]. This tool of assessment includes the risk of bias in the domains of (1) reporting bias, (2) performance bias, (3) detection bias, (4) selection bias, and (5) attrition bias. The studies were judged as low risk of bias, high risk of bias, and unclear risk of bias. Any disagreement was resolved by a further discussion between the authors. The results on the risk assessment of the selected studies are summarised in Figure 3, while the graph in Figure 4 represents the bias assessment.

## 3. Results

### 3.1. Literature Search

**Figure 5:**
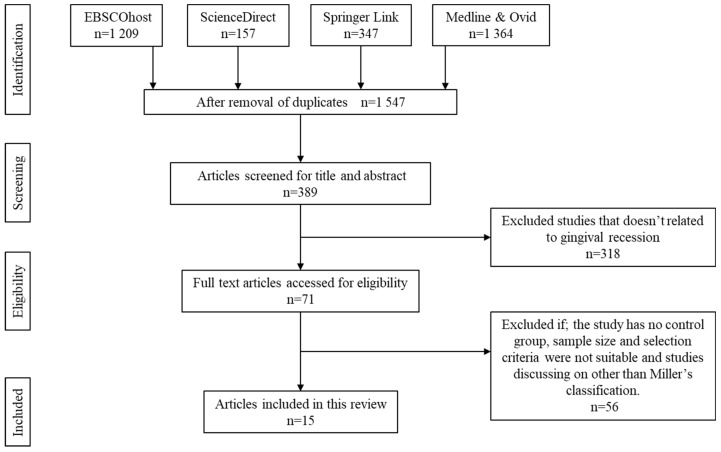
Identification and screening for literature search.

The literature search identified 3077 articles that were considered potentially relevant to be reviewed. Approximately 1530 articles were removed owing to duplication. All of the remaining articles were then screened for titles and abstracts, after which 1158 articles were removed. Another 318 articles were excluded owing to the unmatched content on the physicochemical property of the Col scaffold or the response upon implanting the scaffold towards the GR. From the remaining 71 articles, 56 articles were rejected after full text screening either owing to the absence of a control group, the sample size and selection not meeting the criteria set by this study, and studies focusing on something other than Miller’s classification. A total of 15 articles were retrieved for further analysis. The flow chart of the screening, identification, and the reasons for exclusion are summarised in Figure 5. Data extraction was performed based on the selected articles as shown in Table 1, Table 2 and Table 3.

### 3.2. Bioscaffold Three-Dimensional Structure

Col is effective for healing as it has been categorised as a weak immunogen and highly compatible in humans. The characteristics of ease of manipulation and adaptability further contribute to the development of Col-based scaffolds to be incorporated in GR healing mechanisms. Rosdiani et al. performed a study to identify the appropriate composition of Col-chitosan-glycerol to be used as an alternative treatment for GR. The scaffold designed by the group has an acceptable pore size ranging from 102.4 μm to 143.5 μm, with a thickness in the range of 0.412 mm to 0.515 mm. In addition, the mechanical test (pulling test) results vary from 0.67 MPa, 0.46 MPa, 0.80 MPa, and 2.36 MPa with a percentage of swelling from 1354.04%, 1021.78%, 801.91%, and 413.9% for a Col-chitosan constitution with the ratio of 3:7, 4:6, 5:5, and 6:4, respectively, followed by the addition of 2 mL of glycerol [11]. These findings correlated with another study performed by Ashworth et al., whereby the group in which the pore size is near to 100 μm is ideal for cell migration and proliferation in periodontal application [60]. Beside that, the reduction of Col swelling percentage is possible owing to the presence of a hydrophilic group [43], which is the lysine side [61]. This hydrophilic group has the potential to form a bond with the surrounding solution, thereby contributing to the hydrophilicity characteristics [43].

**Figure 6:**
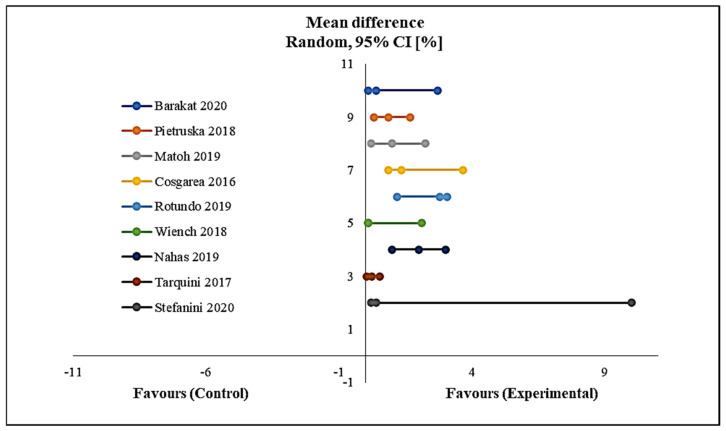
Forest plot shows mean reduction in recession depth. CI, confidence interval.

Using a meta-analysis as shown in Figure 6, the results of the reduction in recession depth indicated a significant reduction from the start to the end of follow-up in favour of the intervention group with *p* < 0.00001. No heterogeneity was detected among the included studies in this meta-analysis (Figure 6).

**Figure 7:**
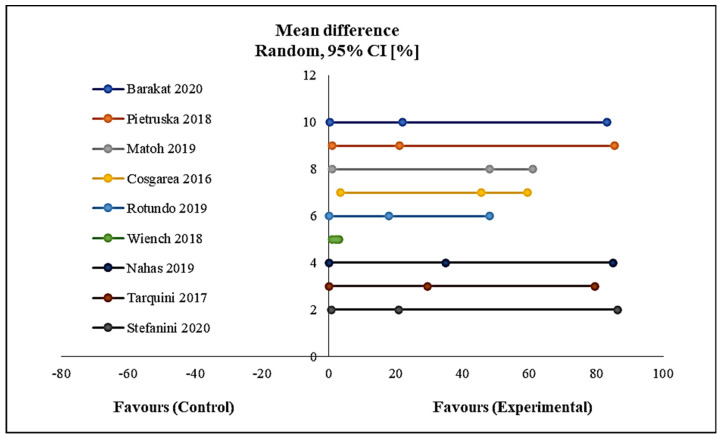
Forest plot shows mean % of mean root coverage.

Using a meta-analysis as shown in Figure 7, the results of the mean percentage of root coverage indicated a significant amount of root coverage from the start to the end of follow-up in favour of the intervention group with *p* < 0.005. No heterogeneity was detected among the included studies in this meta-analysis (Figure 7).

**Figure 8:**
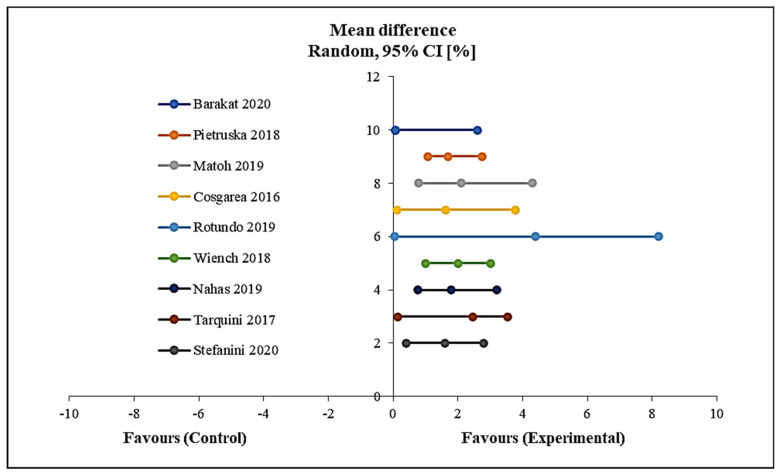
Forest plot showing the mean width of keratinised tissue.

Using a meta-analysis as shown in Figure 8, the results of the mean increase of the width of keratinized tissue indicated a significant increase in the width of keratinised tissue from the start to the end of follow-up in favour of the intervention group with *p* < 0.0001. No heterogeneity was detected among the included studies in this meta-analysis (Figure 8).

### 3.3. Cellular–Bioscaffold Interaction

Nurfriana et al. conducted an in vitro study to evaluate the outcome of Col-chitosan-glycerol scaffold for GR. In this study, the ratio of Col-chitosan was manipulated while the level of glycerol was set as a constant variable at the level of 2 ml for all tests. The group developed a scaffold with a thickness ranging from 0.51 mm to 0.65 mm, which exhibits a pore size between 26.68 μm to 191.7 μm. Col-chitosan with a ratio of 7:3 that has a 2 ml additive of glycerol showed the lowest cytotoxicity (50%) effect. The scaffold completely degraded (100%) on the 14th day with the Col-chitosan ratio of 9:1 [12]. Soheilifar et al.’s study further supported the observation in the previous study by indicating that a complete healing period of gingival ranges from 7 to 14 days [62]. Therefore, the complete scaffold degradation on the 14th day proves that it is suited for the periodontal application. The outcome measured showed that the range of living cell’s percentage for BHK-21 fibroblast cells varies between 4.50% and 6.66% [12]. The results clearly indicated that the developed scaffold is non-toxic for periodontal application, according to the statement by Khoswanto et al. [63]. Meanwhile, the scaffold achieved an equilibrium point after 7 min and the percentage of the swelling dropped drastically after 8 to 10 min [12]. This can be owing to the failure of the hybrid scaffold to bind to the water molecules upon exceeding its limits [64].

### 3.4. The Efficiency of Gingival Tissue Regeneration

Raita et al. conducted a pilot study to assess the response of gingival CT towards three-dimensional (3D) Col nanofibre-coated titanium dental implants. In the study, the Col was sprayed to the screw-type Col implants. The group observed uniformity of Col nanofibre, which is expected to enhance the penetration of gingival collagen fibre into the nanofibre to provide a stronger attachment of gingival CT towards the implant. The histological analysis indicated an absence of adverse events and inflammation. The group discovered that the gingival CT exhibits a direct contact with the implant. Through the study, the group postulated that the 3D environment contributes to the CT elongation surrounding the implants as well as the capacity to control the uniformity of the Col fibres [65]. Similar findings were observed by other researchers, who reported that 3D Col-based environment supports the survival and regeneration of the cells [66,67,68].

Subsequently, Hatayama et al. evaluated the effectiveness of a Col-based scaffold for regeneration of gingiva in beagle dogs by manipulating the pH levels. The thickness level for subjects treated with Col-based scaffold exhibits positive outcomes compared with the control group (no scaffold). Furthermore, rapid gingival tissue formation was observed for Col scaffold at pH 7.4 compared to pH 3.0 [13]. The result indicates that the optimum pH for Col scaffold greatly influences the rate of healing. In relation to this outcome, Nakada et al. stated that Col scaffold at pH 7.4 was able to sustain the mechanical strength, thereby conserving the space for regeneration of the tissue. This is achievable owing to the light denaturation and uniformity of Col fibril arrangement [69]. This statement is further supported by other researchers, who reported that manipulation in pH can greatly influence the physicochemical properties of Col, particularly the porosity, mechanical strength, and adhesion of the cells towards the scaffold [69,70,71]. Hatayama et al. proved that pH 7.4 is the most suitable microenvironment for Col-based scaffold to be assimilated into the periodontal application [69]. 

Another in vivo study was done by Schmitt et al. to compare the efficacy between the porcine-derived Col matrix and subepithelial connective tissue graft (SCTG) for GR. The results were assessed after 10 months of intervention. The thickness of the CT in the SCTG group for five different regions was 1.21 mm, 1.45 mm, 1.46 mm, 1.27 mm, and 1.24 mm, while for the Col group, it was 0.94 mm, 1.12 mm, 1.14 mm, 1.1 mm, and 0.99 mm. Matured blood vessels, papillary indentions, and aggregation of fibroblasts towards Col were extensive in the SCTG group compared with the Col group. In contrast, direct bonding of CT to the periosteum and with a matured Col fibre was observed in both groups. There was no significant difference seen for a vascular endothelial growth factor (VEGF) and Col-I expression in both groups [14]. These findings showed that SCTG outranges Col matrix as the current approved gold standard treatment for GR. However, the Col matrix can be used as a potential alternative treatment for GR, as the Col matrix is a natural component of the human body, thus it can support the regeneration of the periodontal tissue [72,73,74,75].

Nonetheless, Shirakata et al. studied the effect of PADM in GR in comparison with coronally advanced flaps. The group noticed a rapid regeneration of periodontal tissue compared with the control measure. The study demonstrated a dense deposition of Col fibres, increased angiogenesis, and stimulation of VEGF. This proves that the Col composition in PADM is biocompatible for the gingival tissue, which enhances the migration of the cells, thereby contributing to the healing mechanism of GR [15]. Similarly, Cha et al. noticed that a combination of the Col matrix with FGF-2 shows a promising result in the healing of GR. This is because the Col matrix acts as a pro-angiogenic factor that enhances the formation of a new blood vessel at the GR site and is a carrier material for FGF-2. FGF-2 will directly stimulate the differentiation in the osteoblast, leading to the formation of cementum. Being a resorbable material, Col matrix is a perfect biomaterial to treat GR [16].

### 3.5. Clinical Trial Effectiveness

A clinical trial was carried out by Tarquini to test the efficacy of equine Col matrix incorporated with coronally advanced flap for GR. In this study, the groups were initially categorised based on their recession type of either type I or II. After a year of follow-up, those subjects with recession type I intervene with equine Col matrix showed an average root closure of 94.4% with a complete root closure of 88.9%, while those intervened with CT graft showed 95.6% and 90.0%, respectively. In type II recession, the Col matrix group result was 95.8% and 83.3%, while for the CT graft group, it was 93.7% and 75.0%, respectively. The results indicated that Col matrix is more effective for type II recession compared with type I. However, no significant difference was observed in both of the intervene types [23]. In both recession types, Col matrices support the root coverage owing to the native characteristics of the Col matrix as described by Masci et al. and Milinkovic et al. These studies reported that Col enhances fibroblast migration. This eventually aids the reparation of mesenchymal as well as the population of cells responsible for the autologous grafting, thereby contributing to the healing of GR [76,77].

Similarly, Stefanini et al. tested the efficiency of VCMX in addition to coronally advanced flap for GR type I using 10 subjects through clinical trials. The study showed that all patients experience a slight swelling up to day 7, whereas it persists up to day 14 in three of the patients. At the same time, 50% of the patients showed a keloid-like surface at the intervene area, while the rest of the tissue appeared normal. Incomplete closure of the wound was observed in two of the patients. The calculated mean of root coverage, recession depth, recession, probing depth, keratinized tissue, depth, and thickness of gingival shown in Table 1 proved the effectiveness of GR healing, as 80% of the patients showed a complete wound closure [24]. An almost identical result was observed by Thoma et al., who documented 33% failure of complete wound closure upon implanting VCMX for the augmentation of soft tissue at the peri-implant site [70]. This finding was further confirmed by Schmitt et al., showing that porcine Col matrices possess long-term stability and are appropriate for gingiva regeneration [78,79].

Furthermore, a randomised clinical trial conducted by Pietruska et al. showed that the Col matrix was less effective compared with SCTG for GR. The group demonstrated that SCTG is far more superior in decreasing the GR height and width and increasing the thickness of gingival and formation of the keratinized tissue. Simultaneously, SCTG presented >83% of mean root coverage with 45% of complete GR coverage. Conversely, only 53% of mean root coverage and 10% of complete GR coverage were noticed with the Col matrix. It was referenced that the Col matrix and SCTG promote healing in GR upon integration with the coronally advanced tunnel technique. However, there was evidence that SCTG is superior to the Col matrix [25]. In contrast to their conclusion, Cieślik-Wegemund et al. revealed that the Col matrix has less potential in the periodontal recession healing compared with CTG [80].

## 4. Discussion

The data obtained from previous articles indicated a positive outcome of Col for GR healing. In the context of GR healing, the type of recession greatly influences the effect of Col in the healing process. Hence, 100% of the articles analysed in this study support the statement that Col can be used for gingival healing [11,12,13,14,15,16,17,18,19,20,21,22,23,24,25]. However, 13% of the researchers emphasise that Col has less potential for GR healing compared with SCTG [14,18]. Two studies in this review analysed the physicochemical properties of the Col-based scaffold and the effect of the scaffold in GR [11,12]. Both studies demonstrated that the characteristics of scaffold thickness, pore size, swelling ratio, tensile strength, degradation, and cytotoxicity level matched the ideal requirement of scaffold for tissue regeneration [81]. Thus, the designated scaffold manifests positive results in the in vitro testing.

One study concentrates on integrating Col with the means of coating to the existing titanium technique to treat GR. This study proved that the 3D environment of Col is more effective in promoting tissue regeneration compared with the 2D environment. The study also reported that Col nanofibre shows a rapid healing progression compared with the control measures [65]. The outcome of this study was supported by Waddington et al., as they stated that the 3D environment facilitates better migration and proliferation. The group also added that the behaviour of the cells differs tremendously in the 3D environment compared with the 2D environment [82]. Particularly in the dental application, Fraley et al. proved that the 3D environment enhances the regulation of osteoid matrix secretion, thereby imitating the native environment of the tissue [83].

In the context of the functionality of Col scaffold, it has been scientifically proven by Hatayama et al. that pH 7.4 is an ideal pH for optimum functionality for periodontal application [13]. This statement was supported by Ahmadi et al. as the group demonstrated that, in acidic conditions, the necrotic effect is seen in the periodontal tissue and flaps of the mucosa, thereby hindering the normal healing mechanism in the oral tissue [84]. Meanwhile, Murray stated that the alkaline environment is a better option for antibacterial activity and, through mineralisation, pulp healing will be stimulated. However, the group agreed that alkaline-based biomaterial exhibits poor physicochemical characteristics and is highly soluble [85]. In addition, Antoine et al. reported that, under neutral pH, Col fibril will assemble on its own [86], which contributes to the tissue repair mechanism and morphogenesis process [87]. This is essential as the fibril contributes to the tissue contraction that affects the wound closure at the injury site [88]. 

Besides, in the context of the inflammatory response, three researchers reported that Col-based intervene is free from any inflammatory response [13,65,85], while one researcher observed signs of intervened at the inflammation site [25]. Another study reported an infection at the site of peri-implant with an absence of inflammatory response [89]. This is aligned with a report by Castillo-Bricerio et al., whereby the group revealed that Col can trigger inflammatory activity based on the condition of the surrounding environment [90]. Col, upon recognising and binding to the receptor at the injury site, will stimulate the healing factors such as growth factors, cytokines, and matrix metallopeptidase [91]. Stimulation of these factors may result in an inflammatory response, as a part of the tissue healing mechanism. 

From the literature search, a lack of results was identified on the usage of Col scaffold for GR application. Most of the study is deemed to be a generalised application of Col for periodontal healing, particularly concentrating on the Col matrix. There were insufficient studies available for GR healing. Therefore, the mechanism of healing of Col for gingival tissue largely remains unknown. Considering this scenario, more studies are needed in the future as Col is a natural component in the human body and has a high potential to be incorporated in the dental application. In this review, studies reporting on the outcome of the Col scaffold/Col matrix for GR have been discussed.

This article overviews the possible outcome of the Col biomatrix for GR healing. The Col biomatrix has shown superior outcomes compared with the other currently available treatment such as SCTG through in vitro and in vivo models. The Col biomatrix acts similarly to SCTG in terms of reduction in gingival height, width, mean root coverage, complete root coverage, clinical attachment, increased rate of keratinised tissue, and gingival thickness. The designated Col scaffold resembles the ideal scaffold properties in terms of scaffold thickness, porosity, tensile strength, swelling capacity, biodegradation, and absence of cytotoxicity. Hence, Col biomatrix development is tunable according to the need for accelerating the healing phase instead of currently available treatments such as SCTG. Col biomatrix is, therefore, an alternative source of treatment to prevent morbidity on the donor tissue [92]. Nevertheless, various studies have demonstrated that Col naturally possesses the ability to act as a haemostatic agent by recruiting immune cells and skin cells central to the healing mechanism; however, the mechanism is still unclear [93]. Hence, more studies must be done on the Col biomatrix in the future to enable the integration of Col for periodontal healing.

## 5. COVID-19 Outbreak and Dentistry 

Coronavirus disease 2019 or COVID-19 has been a huge challenge in the medical field, particularly in the dental field. In accordance with this statement, the dentist is more prone for this infectious disease compared with general physicians or nurses. This is owing to the direct contact of the dentist with the saliva of the patients, in which a huge load of the virus might reside if the subject is infected [94,95]. Strict regulation must be imposed before handling any procedure in relation to dental checking or surgery. Biomaterial application in the dental application in this challenging period is tough. This is because not only is extra precaution needed, a small technical error can put the subject into the risk of infection because this can be classified as an open wound and the oral cavity has been in constant contact with the environment. As COVID-19 is transmitted through aerosols, any procedure relating to inhalation or dental is not encouraged. In the case of a dental emergency, patients should be handled in an environment in which appropriate care transmission-based precautions are available and complete personal protective equipment for all staffs dealing with the patient is compulsory. Nonetheless, for asymptomatic patients, a detailed history must be taken prior to attending the patient as a protective measure.

## 6. Conclusions

The collagen biomatrix is an alternative choice for GR treatment. It is safe, highly biocompatible, and easy to integrate into the clinical trials owing to the absence of adverse effects. The most beneficial outcome of the utilisation of Col biomaterial for GR is the reduced risk of morbidity. Therefore, the application of Col biomaterial for periodontal could constitute an innovative therapeutic approach as a public health measure to reduce complications and to inculcate a safe approach. This review concludes that Col is a native and widely available biomaterial that is beneficial for tissue healing. All of the articles included in this review demonstrated the positive outcomes of Col for GR healing, either slow or rapid therapeutic effect. However, in some of the clinical trials, Col has been categorised as having less potential for GR. Therefore, further studies are needed to further understand the mechanism of action of Col for GR healing activity. Intervention and cost-effective studies in the context of Col biomatrix application for periodontal in different countries are necessary to evaluate the possible design of public policies in this field.

## 7. Limitation

There are several limitations to this review study. These include the lack of recent studies for Col application in GR. The studies seem to be generalised and no native application of Col scaffold for the periodontal application was found. Furthermore, the in vitro results do not correlate with the clinical trial outcome. There was also no explicit risk of bias checklist found to assess the bias as various parameters were evaluated in the in vivo and in vitro models, and they do not match one another except for in the clinical trials. There was no standardised study design, which makes the observations vary from one study to another.

## 8. Future Trends and Perspective

The current limitation in this study is expected to be overcome in the future. Experimental analysis and clinical trials of Col for GR are being performed to ensure the outcome of Col for GR is being fully explored. In the future, it is expected that Col will be able to be stored as an on-shelf biomaterial that can be used to treat GR, hence no surgery is needed. Thus, the morbidity rate will be greatly reduced. A strict manufacturing procedure and protocols will further ensure the possibility of Col as a perfect substitute for the current risky treatment for GR.

## Figures and Tables

**Table 1:** Cellulose-bioscaffold interaction.

Author	Aim	Study Design	Type of Subject	Findings	Conclusion
Rosdiani et al. [11]	To develop a Col-chitosan-glycerol scaffold for gingival recession	In vitro	Not applicable	-Scaffold thickness ranges from 0.412 mm to 0.515 mm. -Scaffold pore size ranges from 102.4 μm to 143.5 μm. -Tensile strength of the scaffold ranges from 0.46 MPa to 2.36 MPa. -The swelling ratio of the scaffold was recorded at 413.9%, 801.91%, 1021.78%, and 1354.04%. -Addition of Col contributes to the increase of tensile strength.	The developed Col-based scaffold can be used as alternative treatment in means of biomaterial for gingival recession thanks to its excellent physical properties.
Nurfriana et al. [12]	To test the effect of Col-chitosan-glycerol scaffold for gingival recession	In vitro	Not applicable	-The scaffold thickness ranges from 0.51 mm to 0.65 mm. -The pore size of the scaffold ranges from 66.29 μm to 191.7 μm. -Swelling ratio achieved equilibrium point at the 7th minute and starts to reduce from the 8th day onwards. -100% of degradation of the scaffold was observed on day 14. -Addition of Col decreases the tensile value. -Low cytotoxicity level was seen with BHK-21 fibroblast cells. ->50% of cell viability was observed.	The developed scaffold meets the criteria for gingival recession application.

**Table 2:** The in vivo effectiveness of gingival tissue regeneration. GR, gingival recession; CT, connective tissue.

Author	Aim	Study Design	Type of Subject	Findings	Conclusion
Hatayama et al. [13]	To evaluate the efficacy of Col scaffold with different pH for gingival tissue regeneration	In vivo	9 beagle dogs	-Absence signs of inflammation at the wound site. -Increase thickness of submucosal and epithelial tissue was seen upon implanting Col scaffold. -Col scaffold with pH 7.4 shows the highest rate of tissue regeneration.	Col scaffold with a pH of 7.4 is well suited for gingival tissue regeneration.
Schmitt et al. [14]	To compare the effectiveness of porcine Col matrix with a subepithelial connective tissue graft (SCTG) for thickening of gingival	In vivo	8 beagle dogs	-Thickness of CT was recorded in the mean range of 1.06 mm ± 0.27 mm for Col matrix group, while at 1.32 mm ± 0.44 mm for the SCTG group. -Papillary indentation was deeper in the SCTG group compared with the Col matrix group. -Expression of Col-I and VEGF for Col matrix group was 30.57% ± 7.83% and 37.15% ± 9.80%, respectively, while for SCTG, it was 32.64% ± 7.09% and 39.06% ± 7.27%, respectively.	Col matrix is inferior for thickening of gingival soft tissue compared with SCTG.
Shirakata et al. [15]	To compare the effectiveness of coronally advanced flap and porcine acellular dermal matrix (PADM) in GR	In vivo	12 beagle dogs	-PADM shows complete healing with absence of adverse effects in five sites with -PADM shows an increase in soft tissue thickness and height, dense Col fibers, and formation of bone.	PADM is effective in treating Miller class II GR.
Cha et al. [16]	To assess the effectiveness of fibroblast growth factor 2 (FGF-2) incorporated with porcine Col matrix	In vivo	5 mongrel dogs	-After 16 weeks, the recession area was 4.92 ± 1.05 mm^2^. -40% of complete root coverage was seen in the test group. -Increase in soft tissue thickness and formation of bone and cementum was observed in the treated group.	The combination promotes rapid healing of GR.

**Table 3:** Clinical trial effectiveness.

Author	Aim	Study Design	Type of Subject	Follow Up Duration	Findings	Conclusion
Barakat et al. [17]	To evaluate the outcome of Col matrix integrated with coronally advanced flap for treating Miller class II GR	Clinical trial	20 patients	3 month, 6 month, and 12 month	-Mean reduction in recession depth was 0.20 ± 0.37 in the test group. -Increased of probing depth and width of keratinised tissue was 1.42 ± 0.41 and 3.53 ± 0.82, respectively, in the test group.	Combination of Col matrix with coronally advanced flap effective in treating GR and integration of Col matrix prevent the need for secondary surgery.
Matoh et al. [18]	To compare the clinical outcome between CTG and Col matrix in GR patients	Clinical trial	10 patients	6 month and 12 month	-The mean root closure in Col matrix group was 85% ± 24%, while it was 100 in CTG group. -Increase in height of keratinised tissue height is lower in Col matrix compared with the CTG group.	Col matrix is less effective in treating GR compared with the CTG.
Cosgarea et al. [19]	To assess the effectiveness of PADM in Miller class I, II, and III GR	Clinical trial	12 patients	6 month and 12 month	-Improvement of mean root coverage of 73.20 ± 27.71%. -Complete root coverage was >40%. -Reduction in recession depth in maxilla and mandible was 2.51 ± 1.15 and 1.57 ± 1.02, respectively. -Reduction in recession width was 2.92 ± 1.68 and 1.23 ± 1.16 in maxilla and mandible, respectively. -Increase of 0.82 ± 0.72 and 0.87 ± 0.75 for maxilla and mandible, respectively, in attached gingiva was seen. -Keratinised tissue deposition was increase in maxilla and mandible was 0.76 ± 0.51 and 0.62 ± 0.49, respectively. -Probing depth was 1.00 ± 0.00 and 1.20 ± 0.05 for maxilla and mandible, respectively.	PADM is effective for treating Miller class I, II, and III GR.
Rotundo et al. [20]	To evaluate the outcome in combination of Col matrix and coronally advanced flap for GR	Clinical trial	24 patients	3 month, 6 month, 12 month, and 1 year	-Mean reduction in the recession depth was 2.0 ± 0.8 mm in the test group, while it was 2.0 ± 1.1 mm in the control group. -63% of complete root coverage was achieved in the test group. -> 95% increase in gingival thickness was seen in the test group.	-Combination of Col matrix with coronally advanced flap proven effective in treating GR.
Wiench et al. [21]	To evaluate the effectiveness of Col matrix incorporation with coronally advanced flap for Miller class I and II GR	Clinical trial	12 patients	3 month and 6 month	-Mean reduction in recession depth and width was 0.5 ± 0.6 and 1.4 ± 1.2. -Mean reduction in recession area was 0.6 ± 0.8. -Mean increase of 3.4 ± 0.9 in keratinised tissue width. -The mean reduction between cement enamel junction and mucogingival junction was 4.4 ± 1.1. -Average root coverage was 87% with a complete root coverage of 47%.	Col matrix incorporated with coronally advanced flap is effective in treating Miller class I and II GR.
Nahas et al. [22]	To evaluate the outcome of Col matrix vs. CTG in Miller class I GR	Clinical trial	15 patients	3 month, 6 month, and 12 month	-The mean decrease in recession depth and probing depth was 2.7 mm ± 1.1 mm and 1.1 mm ± 0.4 mm, respectively. -The mean increase of keratinised tissue width was 2.2 mm ± 1.0 mm. -Root coverage was >77% with 60% of complete root coverage.	Both Col matrix and CTG promote healing in GR.
Tarquini [23]	To test the efficacy of equine Col matrix incorporated with coronally advanced flap for gingival recession	Clinical trial	50 patients	1 year	-The probing depth, recession depth, and width of keratinized tissue for both are 1.00 mm ± 0.40 mm, 0.15 mm ± 0.37 mm, and 3.38 mm ± 0.57 mm, respectively, for the equine Col matrix group. -For recession type I, the Col matrix group shows keratinized tissue width of 3.56 mm ± 3.50 mm, probing depth of 0.94 mm ± 0.42 mm, and a recession depth of 0.11 mm ± 0.12 mm. -For recession type II, the Col matrix group shows keratinized tissue width of 3.00 mm ± 0.53 mm, probing depth of 1.13 mm ± 0.35 mm, and a recession depth of 0.25 mm ± 0.46 mm. -Average root coverage for equine Col matrix group was 94.2% ± 14.7% with a complete root coverage of 84.6%.	Equine Col matrix can be used to treat GR.
Stefanini et al. [24]	To test the efficiency of volume stable Col matrix (VCMX) in addition of coronally advanced flap for gingival recession	Clinical trial	10 patients	6 month and 12 month	-VCMX possesses the ability to soak up blood with a high elasticity. -20% of the subjects showed incomplete wound closure. -96.7 ± 10.4% of root coverage was recorded. -Recession depth shows a mean range of 0.1 mm ± 0.3 mm. -Recession width was recorded in the mean range of 0.3 mm ± 0.9 mm. -Probing depth mean was seen in the mean range of 1.9 mm ± 0.6 mm. -Keratinized tissue width was observed in the mean range of 2.2 mm ± 0.6 mm. -Thickness of gingival was seen in the mean range of 1.3 mm ± 0.4 mm. -Increase in the VCMX dimension contributes to the closure of the wound. -Absence of bleeding, pus, abscess, and fistula was observed.	Volume stable Col matrix is an effective method to treat gingival recession.
Pietruska et al. [25]	To compare the effectiveness of coronal tunnel technique that has been modified with subepithelial connective tissue (SCTG) and Col matrix for gingival recession	Clinical trial	20 patients	6 month and 12 month	-Gingival recession height in Col matrix side was reduced from 1.95 mm ± 0.76 mm to 0.95 mm ± 0.79 mm, while for SCTG, it was from 1.94 mm ± 0.66 mm to 0.40 mm ± 0.69mm. -Mean root coverage for Col matrix was 53.2%, while for SCTG, it was 83.1%. -20% of complete root coverage was achieved with Col matrix, while it was 67% with SCTG. -Col matrix shows a reduction in recession width from 2.97 mm ± 0.75 mm to 2.08 mm ± 1.30 mm, while in SCTG, it was from 0.76 mm ± 0.31 mm to 1.86 mm ± 0.48 mm. -Col matrix exhibits a statistical different of 1.1 mm with clinical attachment level, compared with SCTG, which is 1.54 mm. -Increased keratinized tissue was seen with Col matrix, from 1.38 mm ± 0.68 mm to 1.91 mm ± 0.84 mm, while for SCTG, it was from 1.28 mm ± 0.72 mm to 4.06 mm ± 1.59 mm. -Gingival thickness for Col matrix side was 1.10 mm ± 0.37 mm, while for SCTG, it was 1.86 mm ± 0.48 mm. -No changes seen in probing depth in either procedure. -10% complete coverage of gingival recession was achieved with Col matrix, while it was 45% for SCTG. -Signs of inflammation with extended healing were seen in two patients.	Col matrix is less effective compared with SCTG for gingival recession.
